# Bioactivity and biomedical applications of pomegranate peel extract: a comprehensive review

**DOI:** 10.3389/fphar.2025.1569141

**Published:** 2025-03-26

**Authors:** Jinsong Du, Heming Wang, Lingyun Zhong, Shujie Wei, Xiaoqiang Min, Hongyan Deng, Xiaoyan Zhang, Ming Zhong, Yi Huang

**Affiliations:** ^1^ School of Health Management, Zaozhuang University, Zaozhuang, China; ^2^ Department of Teaching and Research, Shandong Coal Health School, Zaozhuang, China; ^3^ School of Nursing, Jilin University, Jilin, China; ^4^ School of Pharmacy, Jiangxi University of Chinese Medicine, Nanchang, Jiangxi, China; ^5^ Image Center, Zaozhuang Municipal Hospital, Zaozhuang, China; ^6^ Department of Geriatics, Shandong Healthcare Group Xinwen Central Hospital, Taian, China; ^7^ Magnetic Resonance Imaging Department, Shandong Healthcare Group Zaozhuang Central Hospital, Zaozhuang, China; ^8^ Lanshu Cosmetics Co., Ltd., Huzhou, Zhejiang, China

**Keywords:** pomegranate peel extract, bioactivity, biomedical applications, nanodrug carriers, hydrogels, tissue engineering scaffolds

## Abstract

Pomegranate peel is a by-product generated during the processing of pomegranate (*Punica granatum* L.) fruit, accounting for approximately 50% of the total mass of the fruit. Although pomegranate peel is usually regarded as waste, it is rich in various bioactive metabolites such as polyphenols, tannins, and flavonoids, demonstrating significant medicinal and nutritional value. In recent years, Pomegranate peel extract (PPE) has shown broad application prospects in the biomedical field due to its multiple effects, including antioxidant, anti-inflammatory, antibacterial, anti-apoptotic properties, and promotion of cell regeneration. This review consolidates the major bioactive metabolites of PPE and explores its applications in biomedical materials, including nanodrug carriers, hydrogels, and tissue engineering scaffolds. By synthesizing the existing literature, we delve into the potential value of PPE in biomedicine, the challenges currently encountered, and the future directions for research. The aim of this review is to provide a scientific basis for optimizing the utilization of PPE and to facilitate its broader application in the biomedical field.

## 1 Introduction

Pomegranate (*Punica granatum* L.), belonging to the family Lythraceae and the genus Punica, is one of the oldest edible fruits, originating in Persia (modern-day Iran) and surrounding areas. It was later spread to Asia, North Africa, and the Mediterranean regions through activities such as seafaring and wars, and is now widely cultivated around the world (Benedetti et al., 2023; Langgut, 2024). It is estimated that the total global cultivation area of pomegranate exceeds 300,000 ha, with an annual production of approximately 3 million tons (Melgarejo-Sánchez et al., 2021). Pomegranate peel is one of the main components of the fruit, accounting for about 50% (Arun et al., 2022; Derakhshan et al., 2018). In current pomegranate product processing, pomegranate peel is usually discarded as waste, resulting in around 1.6 million tons of waste annually (Bertolo et al., 2021; Giri et al., 2023). In addition to resource wastage, pomegranate peel waste negatively impacts natural resources such as land and water, further exacerbating environmental issues (Sagar et al., 2018).

Pomegranate peel is not without value but possesses unique medicinal properties. Thousands of years ago, pomegranate peel was widely used in traditional medicine across many cultures (Li et al., 2006; Liu et al., 2012). Historically, pomegranate peel has been utilized in various cultures for medicinal purposes. The Romans employed it as an anthelmintic, while some ethnic groups in the Middle East and South America have used boiled pomegranate peel for treating dysentery (Ge et al., 2021; Longtin, 2003). In traditional Chinese medicine, pomegranate peel is regarded as a potent astringent and anti-inflammatory agent, applied to treat traumatic bleeding, infections, and gastrointestinal disorders such as diarrhea (Mo et al., 2013). In Ayurvedic medicine, it is similarly valued as an effective remedy for oral infections (Rafey et al., 2021). In modern times, the medicinal efficacy of pomegranate peel has been scientifically validated. Studies reveal that pomegranate peel is rich in bioactive metabolites such as tannins, phenolic acids, and flavonoids, which impart a range of pharmacological effects, including antioxidant, anti-inflammatory, antibacterial, and anticancer activities (Ain et al., 2023; Ozkan, 2023; Siddiqui et al., 2024). Research has demonstrated that the antioxidant capacity of pomegranate peel extract (PPE) is nearly ten times greater than that of other parts of the pomegranate fruit, a property closely linked to its polyphenolic structure (Hanafy et al., 2021). Furthermore, PPE is capable of modulating several critical molecular pathways, including mitogen-activated protein kinases, nuclear factor-kappa B (NF-κB), cysteine-aspartic acid-specific proteases, and peroxisome proliferator-activated receptors, thus providing theoretical support for its use in the treatment of a variety of diseases (Andishmand et al., 2025). In addition to its notable pharmacological properties, PPE can be processed into nanodrug carriers, hydrogels, and other biomedical materials through chemical reduction, complexation, emulsification, and hydration methods, offering potential therapeutic applications for diseases such as diabetes and breast cancer (Andishmand et al., 2023; Askari and Nikoonahad Lotfabadi, 2020; Baccarin and Lemos-Senna, 2017; Ganeshkumar et al., 2013; Savekar et al., 2024; Tiyaboonchai et al., 2015). Thus, utilizing pomegranate peel, a by-product, can achieve resource recycling and create new economic value.

**FIGURE 1:**
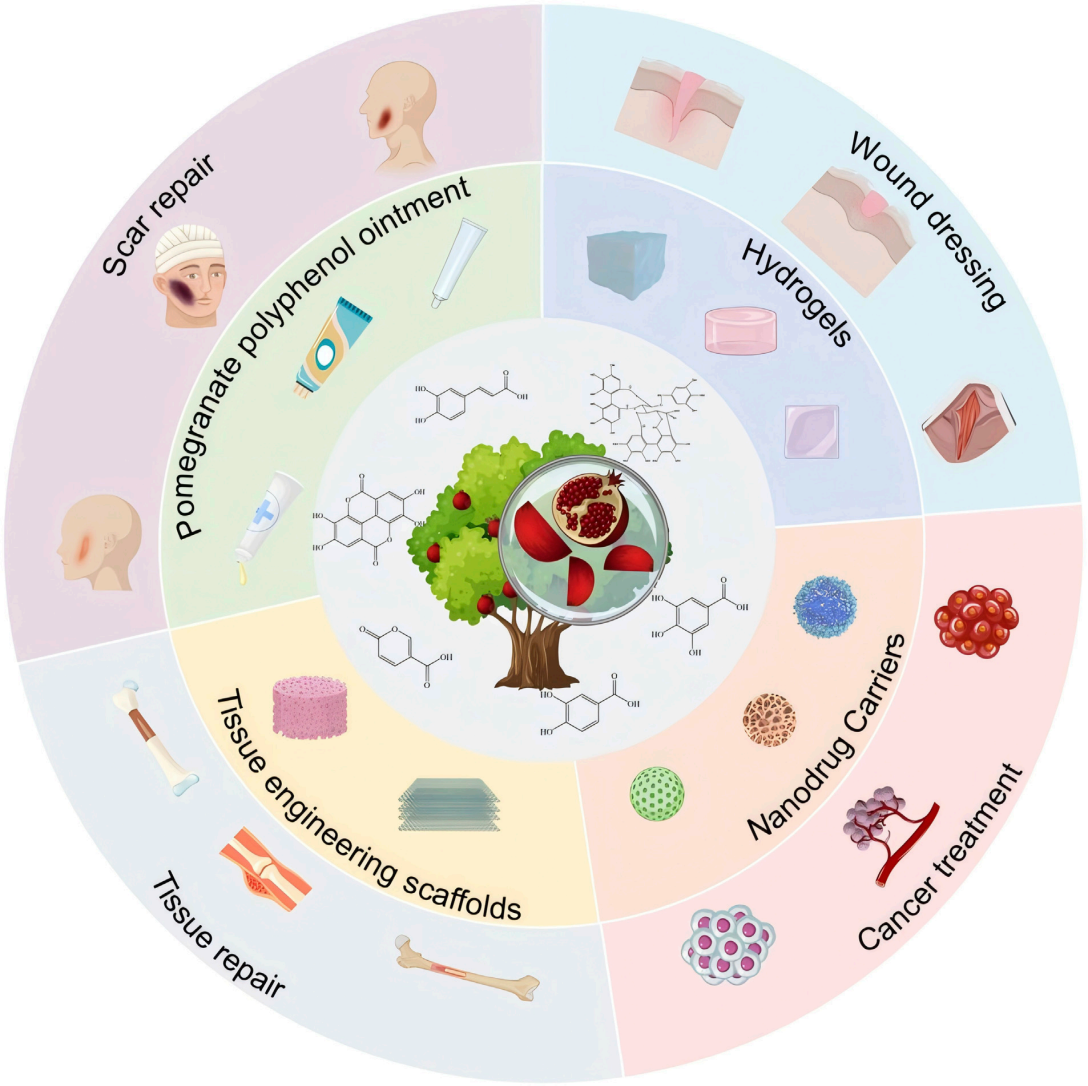
Bioactivity and biomedical applications of pomegranate peel extract.

This paper aims to explore the main bioactive metabolites of PPE and its applications in biomedicine (Figure 1), while providing an in-depth analysis of its limitations in practical applications and its development potential. The goal is to offer a theoretical foundation and scientific support for the future development and utilization of pomegranate peel.

## 2 Retrieval methods

The literature review was conducted using several databases, including Web of Science (https://webofscience.clarivate.cn), ScienceDirect (www.sciencedirect.com), and PubMed (https://www.ncbi.nlm.nih.gov/). The search keywords included “pomegranate peel extract,” “Punica granatum L.,” “bioactivity,” “hydrogels,” “nanodrugs,” “tissue engineering,” “biomedical materials,” and their combinations. All duplicate articles identified during the search were excluded. The species names were verified using the taxonomic database (http://mpns.kew.org/mpns-portal/).

## 3 Bioactive metabolites in pomegranate peel

The content of polyphenolic metabolites in pomegranate peel is significantly higher than in the flesh and seeds, containing ellagic acid, punicalagin, punicalin, and other ellagitannins and their derivatives, as well as gallic acid, coumaric acid, and other phenolic acids, along with flavonoids such as catechin and epicatechin (Table 1). These polyphenolic metabolites exhibit a wide range of bioactivities and can exert certain physiological or pharmacological effects on organisms, including antioxidant, anti-inflammatory, and antibacterial activities (Table 2).

**TABLE 1:** Metabolites in PPE.

	No.	Metabolite	Molecular formula	Structure	References
Ellagitannins	1	Ellagic acid	C_14_H_6_O_8_	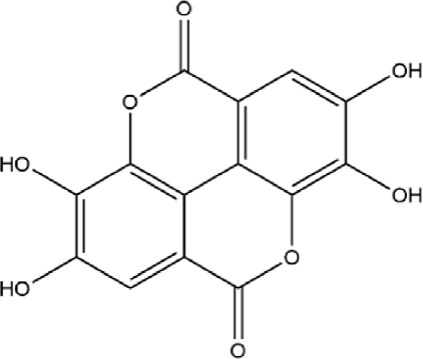	Gaya et al. (2018)
	2	Punicalagin	C_48_H_28_O_30_	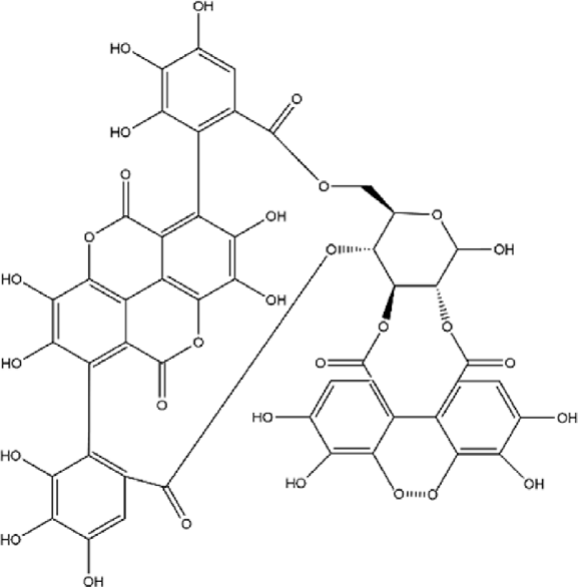	Lyu et al. (2017)
	3	Punicalin	C_34_H_22_O_22_	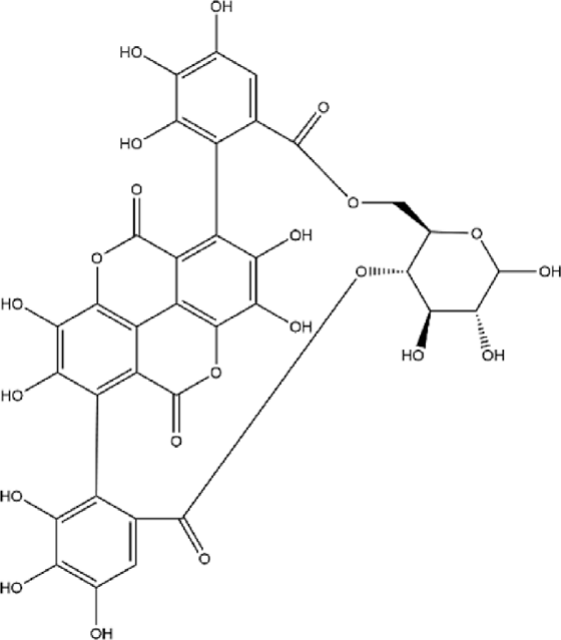	Sun et al. (2017)
	4	Corilagin	C_27_H_22_O_18_	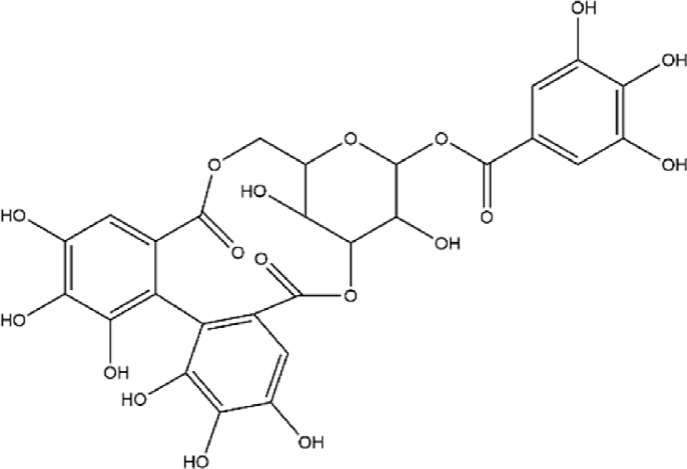	Adili et al. (2020)
	5	Pedunculagin	C_41_H_28_O_26_	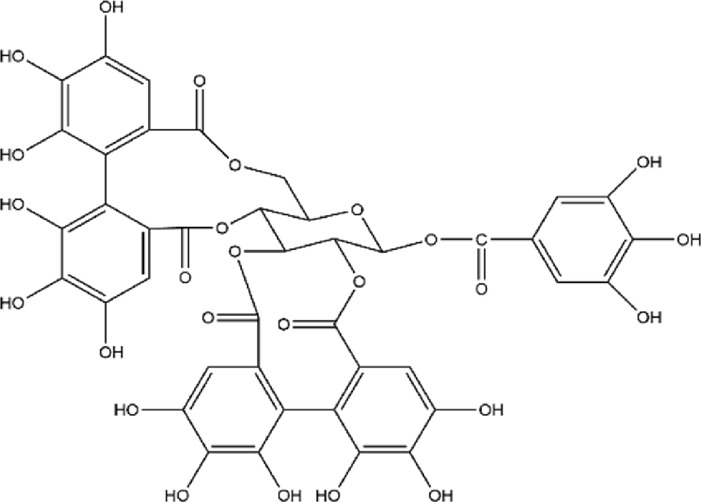	Khan et al. (2017)
	6	Casuarinin	C_41_H_28_O_26_	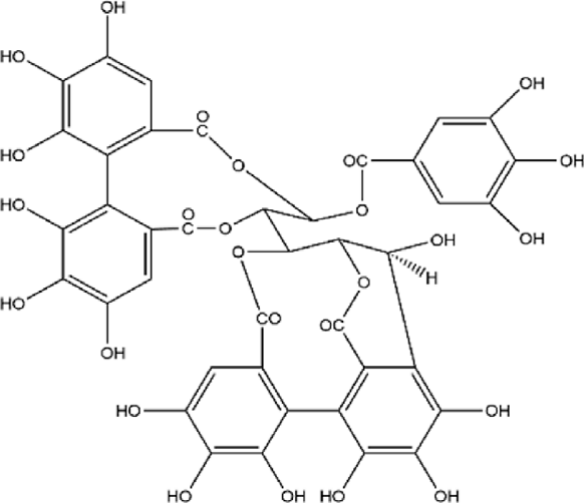	Satomi et al. (1993)
	7	Tellimagrandin	C_41_H_30_O_26_	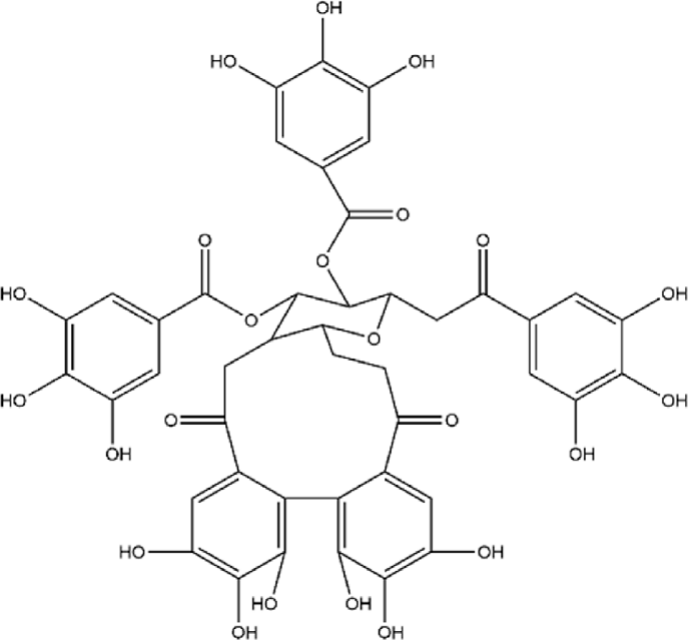	Satomi et al. (1993)
Flavonoids	8	Quercetin	C_15_H_10_O_7_	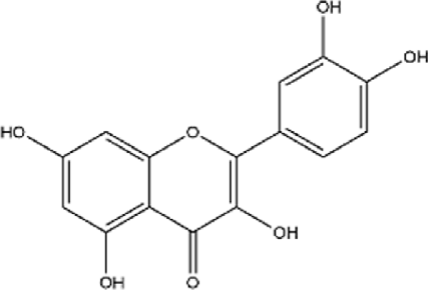	Adili et al. (2020)
	9	Catechin	C_15_H_14_O_6_	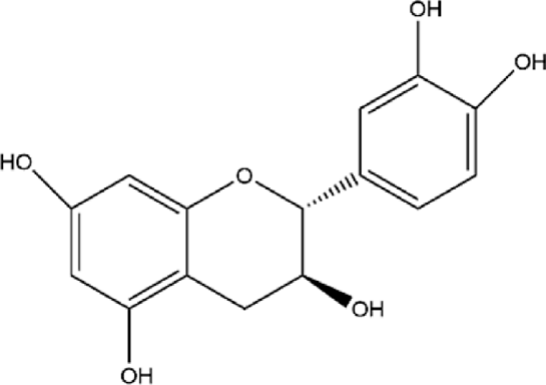	Adili et al. (2020)
	10	Kaempferol	C_15_H_10_O_6_	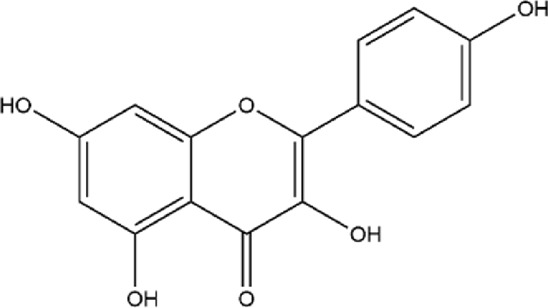	Hussain et al. (2024)
	11	Granatin A	C_34_H_24_O_22_	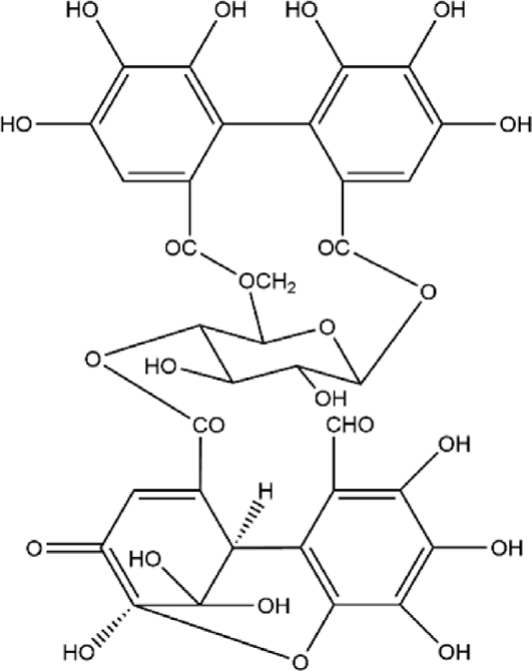	Satomi et al. (1993)
	12	Granatin B	C_41_H_32_O_27_	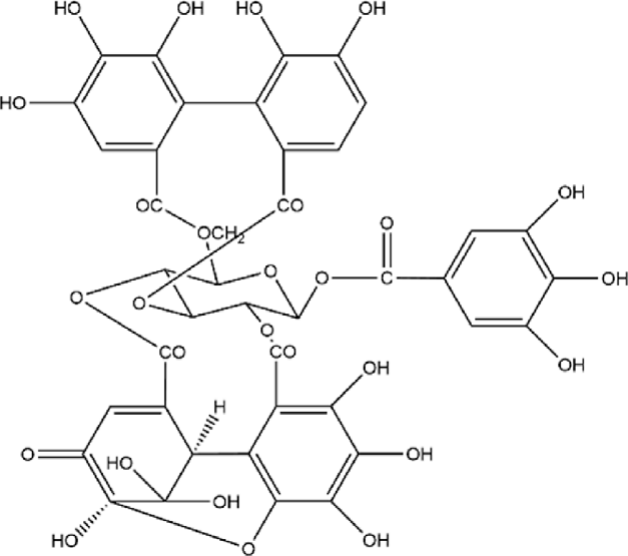	Satomi et al. (1993)
Phenolic Acids	13	Protocatechuic acid	C_7_H_6_O_4_	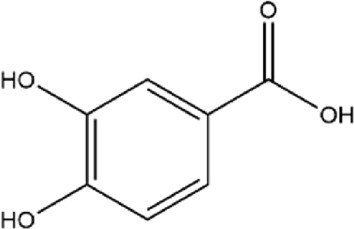	Xiao et al. (2021)
	14	Gallic acid	C_7_H_6_O_5_	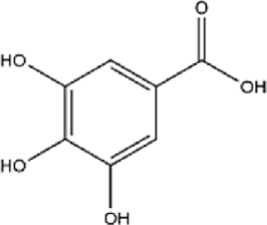	Satomi et al. (1993)
	15	Methyl gallate	C_8_H_8_O_5_	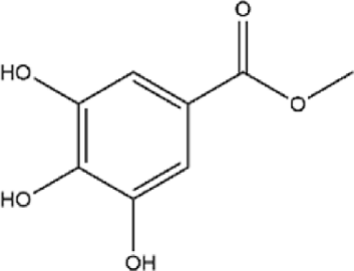	Satomi et al. (1993)
	16	Vanillic acid	C_8_H_8_O_4_	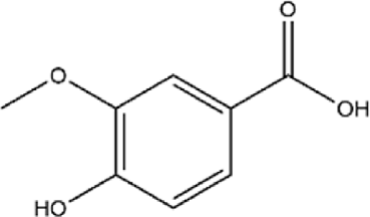	Kumari et al. (2021)

**TABLE 2:** The biological activities of the chemical constituents of PPE.

NO.	Biological activity	PPE	Models	Dosage/Concentration	Conclusion	References
1	Antioxidant	Ellagic Acid	Cisplatin-Induced Nephrotoxicity Model On day 1, adult male Sprague-Dawley rats were intraperitoneally injected with cisplatin (7 mg/kg body weight (mg/kg BW))	From days 1–10, ellagic acid dissolved in corn oil was administered intraperitoneally at a dose of 10 mg/kg BW daily. At the end of the experiment, blood samples were collected from the rats	Ellagic acid exhibited protective effects against cisplatin-induced nephrotoxicity and oxidative stress in rats, potentially due to its potent scavenging activity against superoxide anions and hydroxyl radicals	Ateşşahín et al. (2007)
2			2,3,7,8-Tetrachlorodibenzo-p-dioxin (TCDD)-Induced Nephrotoxicity Model From days 11–13, male Wistar albino rats were injected daily with 15 μg/kg BW of TCDD dissolved in 0.15 mL of corn oil. At the end of the experiment, blood was collected *via* the jugular vein	From days 1–10, ellagic acid was administered orally at a dose of 10 mg/kg BW daily, dissolved in 0.15 mL of 50% dimethyl sulfoxide	Ellagic acid enhanced antioxidant capacity and prevented oxidative stress by maintaining Ca^2+^ homeostasis in HepG2 cells and inhibiting CYP1A1 activity, thereby providing protection against TCDD-induced nephrotoxicity	Padma et al. (2014)
3			High-Fat and High-Cholesterol Diet Model From weeks 1–8, 4-6-week-old New Zealand white rabbits with an average body weight of 1.5 kg were fed a diet enriched with 10% corn oil and 0.5% cholesterol	During the same period, rabbits were supplemented with 1% (w/w) ellagic acid. At the end of week 8, blood samples were collected *via* cardiac puncture	Ellagic acid demonstrated the ability to scavenge RO⋅ or ROO⋅ free radicals, inhibit the production of hydrogen peroxide and singlet oxygen, and suppress lipid peroxidation, thereby improving atherosclerosis	Yu et al. (2005)
4		Punicalagin	Lipopolysaccharide-induced testicular oxidative stress injury model: Day 1–7, adult male ICR mice were injected daily with lipopolysaccharide (600 μg/kg BW)	Day 1–7, mice were orally administered punicalagin at a dose of 9 mg/kg BW daily *via* gavage. On the last day of treatment, male mice were exposed to female mice for 4 days, followed by dissection and examination	Punicalagin significantly reduced the production of the strong oxidant NO and mitigated oxidative stress in the testes by activating nuclear factor erythroid 2-related factor 2 (Nrf2), reducing oxidative damage, and increasing sperm count	Rao et al. (2016)
5			CCl_4_-induced liver injury model: In the 5th week, mice were intraperitoneally injected with 0.07% CCl4 (v/v, 0.1 mL/100 g), and blood and liver tissue samples were collected 16 h later	After a 1-week adaptation period, mice were gavaged daily with punicalagin dissolved in distilled water (80 mg/kg BW) for 4 consecutive weeks	Punicalagin alleviated oxidative stress by increasing liver superoxide dismutase (SOD), glutathione peroxidase (GPx) activity, and Nrf2 protein expression, thereby protecting against CCl_4_-induced liver injury	Luo et al. (2019)
6		Quercetin	3-Nitropropionic acid (3-NPA)-induced oxidative stress model: Day 1–42, SPF-grade female mice were injected daily with 3-NPA (20 mg/kg BW)	Day 1–42, mice were intraperitoneally injected with quercetin (200 mg/kg BW)	Quercetin alleviated oxidative stress in this model by promoting SIRT1 expression, activating the SIRT1/ROS/AMPK signaling pathway, inhibiting ROS accumulation induced by oxidative stress, regulating autophagy levels, reducing cell apoptosis, and ultimately restoring ovarian function	Duan et al. (2024)
7			Xenoestrogen bisphenol S (BPS)-induced behavioral change model in zebrafish: From day 1 to day 21, adult zebrafish were placed in a 15-L aquarium with a BPS concentration of 20.52 M	From day 1 to day 21, quercetin was added to the aquarium water at a concentration of 2.96 M	Quercetin alleviated oxidative stress and behavioral changes induced by BPS by increasing GSH levels and superoxide dismutase activity, while reducing elevated lipid peroxidation in the zebrafish brain	Ragunath et al. (2022)
8		Catechin	Chronic unpredictable mild stress (CUMS) depression model constructed by external stressors: From week 1 to week 8, male Sprague Dawley rats were subjected daily to various stressors such as food deprivation, water deprivation, tail clipping, electric shock, and forced swimming	From week 1 to week 8, catechin hydrate (50 mg/kg BW) was orally administered daily	After catechin treatment, antioxidant parameters such as catalase, glutathione, and superoxide dismutase levels were restored, reversing CUMS-induced depression in rats by alleviating oxidative stress	Rai et al. (2019)
9		Protocatechuic acid	Isoproterenol-induced heart failure model in mice: From day 1 to day 14, C57BL/6NTac mice were intraperitoneally injected with isoproterenol (80 mg/kg BW) to construct a heart failure model	From day 6 to day 14, mice were injected daily with protocatechuic acid (100 mg/kg BW)	Kynurenine-3-monooxygenase (*Kmo*) was identified as a potential target of protocatechuic acid. Protocatechuic acid prevented heart failure by reducing ROS production induced by isoproterenol both *in vivo* and *in vitro* through *Kmo* downregulation	Bai et al. (2023)
10		Gallic acid	3-Nitropropionate acid (3-NP)-induced ovarian oxidative stress model in mice: From day 7 to day 16, female ICR mice were intraperitoneally injected with 3-NP (12.5 mg/kg BW) twice daily	Mice were administered gallic acid *via* oral gavage	Gallic acid exerted its protective effects by increasing antioxidant enzyme activity and reducing the mRNA expression levels of pro-apoptotic genes (*Bim* and *Caspase-3*), thereby inhibiting apoptosis in ovarian granulosa cells	Li et al. (2017)
11	Anti-inflammatory	Ellagic acid	Acute liver injury model induced by Lipopolysaccharide/d-Galactosamine (LPS/GalN) in Mice: Mice were intraperitoneally injected with GalN (800 mg/kg BW) and LPS (50 μg/kg BW), dissolved in PBS. After treatment, the mice were sacrificed	One hour prior to LPS/GalN administration, ellagic acid was injected intraperitoneally	Ellagic acid significantly suppressed the production of pro-inflammatory cytokines such as TNF-α and effectively inhibited Nuclear Factor-kappa B (NF-κB) activation by preventing the phosphorylation of IκB-α and NF-κB p65. This reduced inflammation and prevented liver injury	Gu et al. (2014)
12		Punicalagin	Pelvic inflammatory disease (PID) model in rats: A PID model was constructed by implanting a mixed microbial solution of *Escherichia coli* and *Staphylococcus aureus* into the rat cervix. On days 1–7, female Sprague Dawley rats were injected with 10 mg of progesterone. On days 9, 11, 13, and 15, gelatin sponges soaked in a nutrient broth containing *E. coli* and *S. aureus* were implanted into the cervical region	Rats were divided into preventive and therapeutic groups. The preventive group was administered punicalagin *via* gavage 1 day before PID induction, while the therapeutic group received punicalagin *via* gavage 1 day after confirming the PID model	In both groups, punicalagin significantly reduced IL-1β levels, lipid peroxidation, and catalase activity. It also decreased local leukocyte infiltration and oviduct fibrosis, demonstrating its potential as a safe and effective treatment for PID.	Zoofeen et al. (2024)
13			Collagen-induced arthritis (CIA) model in mice: On day 1, DBA/1 male mice were injected intradermally at the base of the tail with 200 mg of bovine type II collagen emulsified in a 1:1 ratio with complete Freund’s adjuvant to induce an initial immune response. On day 22, an immunization booster was administered with bovine type II collagen emulsified in incomplete Freund’s adjuvant (1:1)	From day 23 to day 37, punicalagin (50 mg/kg BW) was intraperitoneally injected daily	Punicalagin significantly reduced serum levels of IL-6 and TNF-α in CIA mice, alleviated cellular infiltration, synovial hyperplasia, cartilage destruction, and limb swelling, showing strong anti-arthritis effects	Huang et al. (2021)
14		Corilagin	Acute lung injury (ALI) model induced by Lipopolysaccharide (LPS) in mice: C57BL/6 mice were intratracheally instilled with LPS (2.5 μg/g BW) dissolved in PBS.	After 30 min of LPS stimulation, corilagin (10 mg/kg BW) was administered, and the mice were anesthetized and sacrificed 6 h later	Corilagin significantly reduced inflammatory cell infiltration in lung tissues and inhibited the production of pro-inflammatory cytokines TNF-α, IL-6, and IL-1β, thereby providing effective protection against LPS-induced ALI.	Liu F. C. et al. (2022)
15		Quercetin	Osteoarthritis (OA) model induced by monosodium iodoacetate (MIA) in rats: On day 1, male Sprague-Dawley rats were given a single intra-articular injection of 1 mg MIA into the right knee joint	From day 1 to day 28, rats were gavaged with quercetin daily	Quercetin significantly reduced serum levels of inflammatory cytokines, including IL-5, IL-6, IL-7, IL-10, and TNF-α, in OA rats. It also inhibited the expression of MMP-3, MMP-13, ADAMTS4, and ADAMTS5, reducing cartilage matrix degradation and demonstrating excellent anti-inflammatory and chondroprotective effects	Wang et al. (2023)
16		Catechin	Establishment of a coronary heart disease (CHD) model in rats using a high-fat diet and pituitrin injection: From weeks 1–10, 10-week-old SPF-grade Wistar rats were fed a high-fat diet. Forty-eight hours after the last feeding, the rats were intraperitoneally injected with pituitrin (30 U/kg BW) once daily for two consecutive days	Following the pituitrin injections, the rats were administered catechin *via* gavage once daily for 4 weeks	catechin significantly reduced levels of inflammatory biomarkers and cytokines such as C-reactive protein, Lp-PLA2, IL-6, and TNF-α. It also activated anti-inflammatory and protective signaling molecules such as Farnesoid X Receptor, STAT-3, and PKB/Akt, effectively improving the pathological state of CHD.	Tu et al. (2018)
17		Kaempferol	Establishment of a mouse asthma model using ovalbumin (OVA): On days 1 and 15, BALB/c mice were subcutaneously injected with 20 μg of OVA to induce sensitization. From days 29–31, the mice were exposed to 5% OVA aerosol for 20 min daily to establish the asthma model	During days 29–31, the mice were orally administered 0.1 mL of kaempferol solution (10 or 20 mg/kg BW) daily	Kaempferol inhibited the production of macrophage inflammatory protein-2 and the overexpression of its receptor CXCR2. It also modulated the Tyk2-STAT1/3 signaling pathway in response to IL-8, thereby alleviating airway inflammation	Gong et al. (2013)
18		Methyl gallate	Establishment of a hyperuricemia nephropathy (HN) model in mice using potassium oxide (PO): From days 1–28, male C57BL/6 mice were intraperitoneally injected daily with 300 mg/kg BW of PO.	Thirty minutes prior to each PO injection, the mice were administered methyl gallate *via* gavage	Methyl gallate significantly reduced levels of NLRP3, ASC, IL-1β, and caspase-1 proteins in HN mice. It effectively inhibited the activation of the NLRP3 inflammasome, thereby exerting renal protective effects against PO-induced HN.	Liu et al. (2021)
19	Antibacterial	Ellagic acid	Establishment of a mouse *H. pylori* Infection Model using *H. pylori Strain* (Sydney Strain 1): To establish the model, male C57BL/6 mice were orally administered 0.2 mL of bacterial suspension (10^8^ CFU/mL) three times at 2-day intervals using a gavage tube	Two weeks after bacterial infection, the mice were administered ellagic acid (10 mg/kg BW) daily for 1 week *via* gavage	Ellagic acid restored and repaired gastric mucosal damage caused by *H. pylori*, demonstrating significant anti-*H. pylori* effects	De et al. (2018)
20			Establishment of a mouse *Aeromonas hydrophila* infection model: Male MF1 albino mice were fed a bacterial suspension of *Aeromonas hydrophila* (0.2 mL, 2 × 10^8^ CFU) once a week for 2–4 weeks	The mice were administered ellagic acid *via* a stainless-steel gavage tube three times weekly (total dose 150 mg/kg BW) for 2–4 weeks	Ellagic acid treatment significantly increased levels of anti-LPS and anti-ECP IgM in infected mice, maintained the integrity of intestinal villi, and demonstrated antibacterial activity against *Aeromonas hydrophila*	Abuelsaad et al. (2013)
21		Kaempferol	An infection model was established by intraperitoneally inoculating C57BL/6 female mice with 5 × 10^6^ CFU of the wild-type *Listeria monocytogenes EGD* strain	After infection, the mice were treated with kaempferol (100 mg/kg BW)	Kaempferol treatment significantly reduced the bacterial load in major target organs, such as the spleen, and improved the survival rate of infected mice by more than 20% on the 6th day post-infection, demonstrating strong anti-*Listeria monocytogenes EGD* activity	Wang et al., 2022)
22		Quercetin	*Citrobacter* rodentium-induced colitis model in mice: The colitis model was constructed by orally administering 2 × 10^8^ CFU of *Citrobacter rodentium* culture to C57BL/6 mice, which were sacrificed on the 7th day post-infection	During weeks 1–2, quercetin (30 mg/kg BW) was supplemented in the mice’s basal rodent diet.	The mice showed no signs of colitis, and the colon’s bacterial populations, including *Fusobacterium* and *Enterococcus*, were suppressed, indicating that Quercetin exhibits antibacterial activity against *Citrobacter rodentium*	Lin et al. (2019)
23		Gallic acid	*S. Typhimurium* peritoneal infection model in mice: Mice were peritoneally infected with 1 × 10^6^ CFU of *S. Typhimurium*	Starting 3 days before infection and continuing for 10 days after infection, mice were orally administered 100 μL of gallic acid (100 μg/mL BW) daily	Gallic acid treatment significantly increased the levels of cytokines such as IFN-γ, indicating that it might stimulate the host immune response. Infected mice showed a significant reduction in bacterial loads in organs such as the liver and a decrease in mortality, suggesting that the virulence of lethal-dose *Typhimurium* was attenuated. These results indicate that gallic acid effectively protects macrophages and mice from *Typhimurium* infection	Reyes et al. (2017)
24			*B. abortus* peritoneal infection model in mice: Mice were peritoneally infected with 2 × 10^4^ CFU of *B. abortus*	Starting 3 days before infection and continuing for 14 days after infection, mice were orally administered 100 μg/mL BW of gallic acid daily	Gallic acid interfered with *B. abortus* invasion by inhibiting F-actin polymerization and downregulating mitogen-activated protein kinases (MAPKs). Additionally, it induced the secretion of protective cytokines such as IL-12, triggering a protective immune response and inhibiting bacterial proliferation in the spleen, demonstrating its antibacterial activity	Reyes et al. (2018)
25			*Galleria mellonella* (*G. mellonella*) infection model with multidrug-resistant *escherichia coli*: A multidrug-resistant *Escherichia coli* infection model was established by injecting 10 μL of a 2 × 10^6^ CFU/mL bacterial suspension into the right fourth leg of *G. mellonella* larvae	One hour after infection, 10 μL of gallic acid was injected into the left fourth leg of the larvae	Gallic acid disrupted the bacterial outer and inner membranes and inhibited the mRNA expression of membrane permeability-related genes (e.g., acrA, acrB, tolC, acrD, and acrF), significantly improving the survival rate of *Galleria mellonella*. This demonstrates its antibacterial activity against multidrug-resistant *Escherichia coli*	Tian Q. et al. (2022)
26		Methyl gallate	An infection model was constructed by injecting 5 × 10^5^ CFU of extensively drug-resistant *P. aeruginosa* into the hind leg of *Galleria mellonella* larvae	Two hours post-infection, the larvae were injected with methyl gallate	Methyl gallate significantly downregulated the expression of quorum-sensing-related genes in *P. aeruginosa* and dose-dependently improved the survival rate of the larvae. This demonstrates its antibacterial activity against extensively drug-resistant *P. aeruginosa*	Flores-Maldonado et al. (2024)

### 3.1 Ellagitannins

Ellagitannins are derived from esters of hexahydroxydiphenic acid, which undergo hydrolysis to form ellagic acid, and bind with polyols. Through oxidative coupling reactions of ellagic acid moieties, ellagitannins form hexahydroxydiphenyl groups, which are dehydrodimeric ellagic acid esters with aromaticity (Molnar et al., 2024). This property gives ellagitannins a more complex structural characteristic compared to ordinary tannic acids. Ellagitannins in PPE include ellagic acid, punicalagin, punicalin, corilagin, and pedunculagin, among others. Punicalagin, characterized by a high total phenolic content, is the primary contributor to the antioxidant properties of pomegranate products (Nuncio-Jáuregui et al., 2015; Valero-Mendoza et al., 2023). Its antioxidant capacity is remarkably high, approximately 50%, whereas ellagic acid demonstrates a weaker antioxidant capacity, with only 3% per molecule (Shabir et al., 2024). Punicalagin contains galloyl groups esterified with glucose, which undergo hydrolysis to form ellagic acid and punicalin (Yamada et al., 2018). Punicalin retains significant antioxidant activity, effectively scavenging free radicals and reducing oxidative damage to cells, thereby contributing to the prevention of aging and related diseases (Magangana et al., 2020; Ranjha et al., 2023; Sharma et al., 2022).

The role of ellagitannins and their derivatives in disease prevention is closely tied to their antioxidant potential, which increases with the number of hydroxyl groups in their molecular structure (Gupta et al., 2019; Ismail et al., 2016). Pedunculagin, the most abundant and biologically active ellagitannin in pomegranate, has therapeutic effects on inflammatory diseases (Shabir et al., 2024; Xu et al., 2022). Seo et al. used a lupus-prone mouse model with spontaneously occurring severe rheumatoid disease to investigate the effects of different doses of pedunculagin (Seo et al., 2020). As the concentration of pedunculagin increased, the severity of renal damage in the mice significantly decreased. The study demonstrated that pedunculagin could alleviate lupus nephritis in mice by inhibiting the protease-activated receptor 2 pathway. Future research could further explore the role of potential pathways such as NF-κB or MAPK, and evaluate the potential toxic side effects of pedunculagin on the liver and kidneys. Nguyen et al. stimulated human placental, visceral fat, and subcutaneous fat tissue explants with tumor necrosis factor (TNF), and the study showed that punicalagin significantly inhibited the TNF-induced expression of pro-inflammatory factors (IL-1β, IL-6) in human placenta and adipose tissue (Nguyen-Ngo et al., 2020). A strength of this study was the use of human-derived tissues *in vitro*, providing greater clinical relevance. However, the limitation of this research lies in the lack of *in vivo* studies with animal models, leading to insufficient validation of its *in vivo* efficacy and mechanisms. Furthermore, other ellagitannins such as corilagin, casuarinin, and tellimagrandin, which contain multiple hydroxyl groups, exhibit significant antioxidant activity. Corilagin inhibits the reverse transcriptase activity of RNA tumor viruses, thereby demonstrating anticancer effects (Li et al., 2018). Casuarinin exhibits antiviral activity by inhibiting the adhesion and penetration of herpes simplex virus type 2 (Cheng et al., 2002). Chang et al.‘s study showed that tellimagrandin inhibits the activity of methicillin-resistant *Staphylococcus aureus* with a minimum inhibitory concentration of 128 μg/mL (Chang et al., 2019). Overall, ellagitannins and their derivatives possess multiple bioactivities, including antioxidant, anti-inflammatory, antibacterial, and antitumor effects, demonstrating broad application potential in disease prevention and treatment.

**FIGURE 2:**
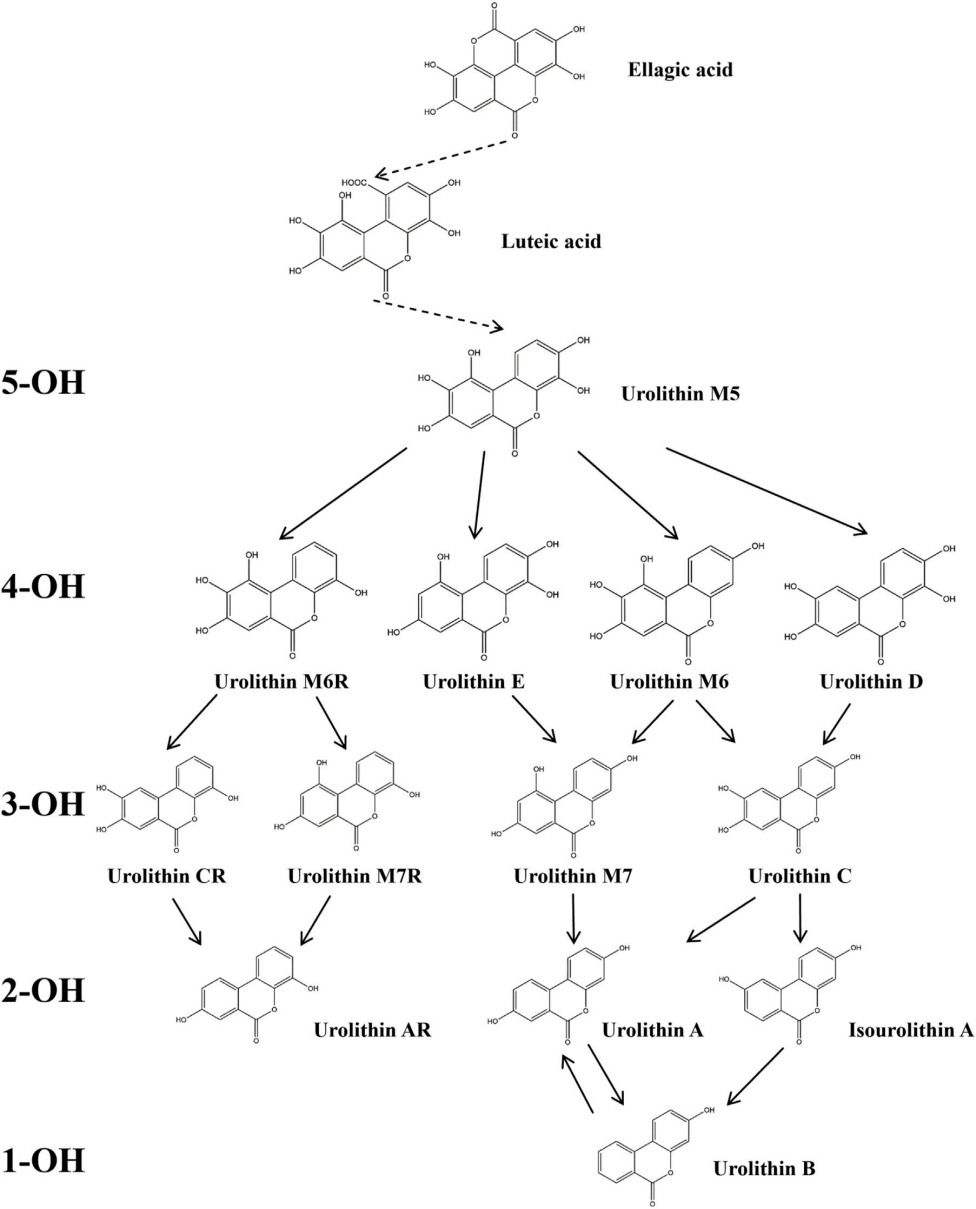
Degradation Pathway of ellagic Acid Metabolism into Uros. In the figure, Uros are classified based on the number of hydroxyl groups (5-OH, 4-OH, 3-OH, 2-OH, 1-OH), and the final products include urolithin A, urolithin B, and Isourolithin A (Xiang et al., 2022).

The metabolic pathways of ellagitannins *in vivo* show significant differences from those of tannic acids. Ellagitannins are not directly absorbed into the bloodstream but are metabolized through hydrolysis processes (Shabir et al., 2024). For example, metabolites such as punicalagin and corilagin are hydrolyzed under acidic or alkaline conditions to generate hexahydroxydiphenoyl (HHDP) groups, which spontaneously lactonize into ellagic acid (Evtyugin et al., 2020). Ellagic acid, a major metabolite of pomegranate peel (Kumar et al., 2022), is a polyphenolic dilactone that has been proven to exhibit various bioactivities, including antioxidant, anti-inflammatory, and antibacterial effects (Cuevas-Magana et al., 2022; Yu et al., 2023). After ingesting ellagitannins or ellagic acid, the human body metabolizes these metabolites through intestinal microbiota, producing 6H-dibenzo [b,d]pyran-6-one derivatives, also known as urolithins (Uros) (Figure 2), which are beneficial to health (Cerdá et al., 2003a; Cerdá et al., 2003b). Depending on the number and position of hydroxyl groups, Uros can be further classified into different types, such as urolithin A, urolithin B, urolithin C, *etc.* Long-term intake of ellagitannin-rich foods can increase the concentration of Uros in bodily fluids such as plasma and urine (Singh et al., 2022; Zhang et al., 2021; Zhang et al., 2020), as well as in tissues like the breast and bones (Andreux et al., 2019; Angeles Avila-Galvez et al., 2019). Research has shown that urolithins play a significant role in the biological activities within the body. For instance, in an inflammatory bowel disease model (Larrosa et al., 2010), following the administration of pomegranate extract, inflammation markers such as inducible nitric oxide synthase (iNOS), cyclooxygenase-2 (COX-2), and prostaglandin E synthase (PTGES) were significantly reduced in the inflammatory rats. Additionally, oxidative stress levels in the plasma and colonic mucosa decreased, and the concentration of urolithins in fecal extracts was significantly lower than in the healthy rat group. In a Parkinson’s disease model, after the administration of pomegranate extract, urolithins were detected in the brains of the affected rats, and improvements were observed in their olfactory and motor impairments (Kujawska et al., 2021). Furthermore, the levels of α-synuclein oligomers were also reduced (Kujawska et al., 2020). Additionally, urolithins can regulate cholesterol metabolism by reducing the abundance of Coriobacteriaceae bacteria and altering the bile acid pool, thereby promoting lipid absorption in the intestines and providing cardiovascular protection (Cortes-Martin et al., 2024). These findings highlight that foods rich in ellagic acid can positively impact health both in the gastrointestinal tract and systemically through urolithins.

Upon entering the human body, ellagitannins undergo bidirectional modulation with the gut microbiota. The gut microbiota can metabolize ellagitannins into urolithin metabolites through hydrolysis, reduction, and other reactions, while ellagitannins and their metabolites can also selectively regulate the composition of the microbiota (Caballero et al., 2022; Henning et al., 2022; Sanchez-Terron et al., 2024). For example, punicalagin intervention significantly enriched short-chain fatty acid-producing bacteria such as *Akkermansia muciniphila* and *Ruminiclostridium 9* in the intestines of high-fat diet mice (Gao et al., 2024), while also increasing the abundance of Lachnospiraceae *NK4A136* and *Bifidobacterium*, which have bile acid metabolism functions (Liu et al., 2024). The gut microbiota, as a “metabolic regulatory organ,” plays a crucial role in the bile acid metabolic cycle. Approximately 95% of primary bile acids are reabsorbed through the terminal ileum sodium-dependent bile acid transporter and enter the enterohepatic circulation, while the remaining 5% are metabolized by bile salt hydrolases and 7α-dehydroxylases secreted by *Bacteroides*, *Clostridium*, *Bifidobacterium*, and *Lactobacillus* into secondary bile acids such as deoxycholic acid and lithocholic acid (Begley et al., 2005; Ridlon et al., 2016). Secondary bile acids play a pivotal role in inflammation regulation. Studies have shown that the levels of secondary bile acids in the intestines of patients with inflammatory bowel disease are lower compared to healthy controls (Lloyd-Price et al., 2019). Supplementation with secondary bile acids can alleviate inflammation in acute and chronic colitis models in mice (Sinha et al., 2020), and remodeling the gut microbiota to improve bile acid metabolism can reduce colitis (Zhou et al., 2023). These findings suggest that ellagitannins may modulate disease development through the gut microbiota-bile acid axis. The bidirectional regulation of ellagitannins and the gut microbiota offers a new perspective on their potential health benefits. However, current research still faces certain limitations, particularly the lack of systematic studies on the combined administration of ellagitannins with specific probiotics. Future in-depth exploration in this area may not only help elucidate their synergistic mechanisms but also open new avenues for the application of PPE in functional nutritional supplements.

### 3.2 Flavonoids

**FIGURE 3:**
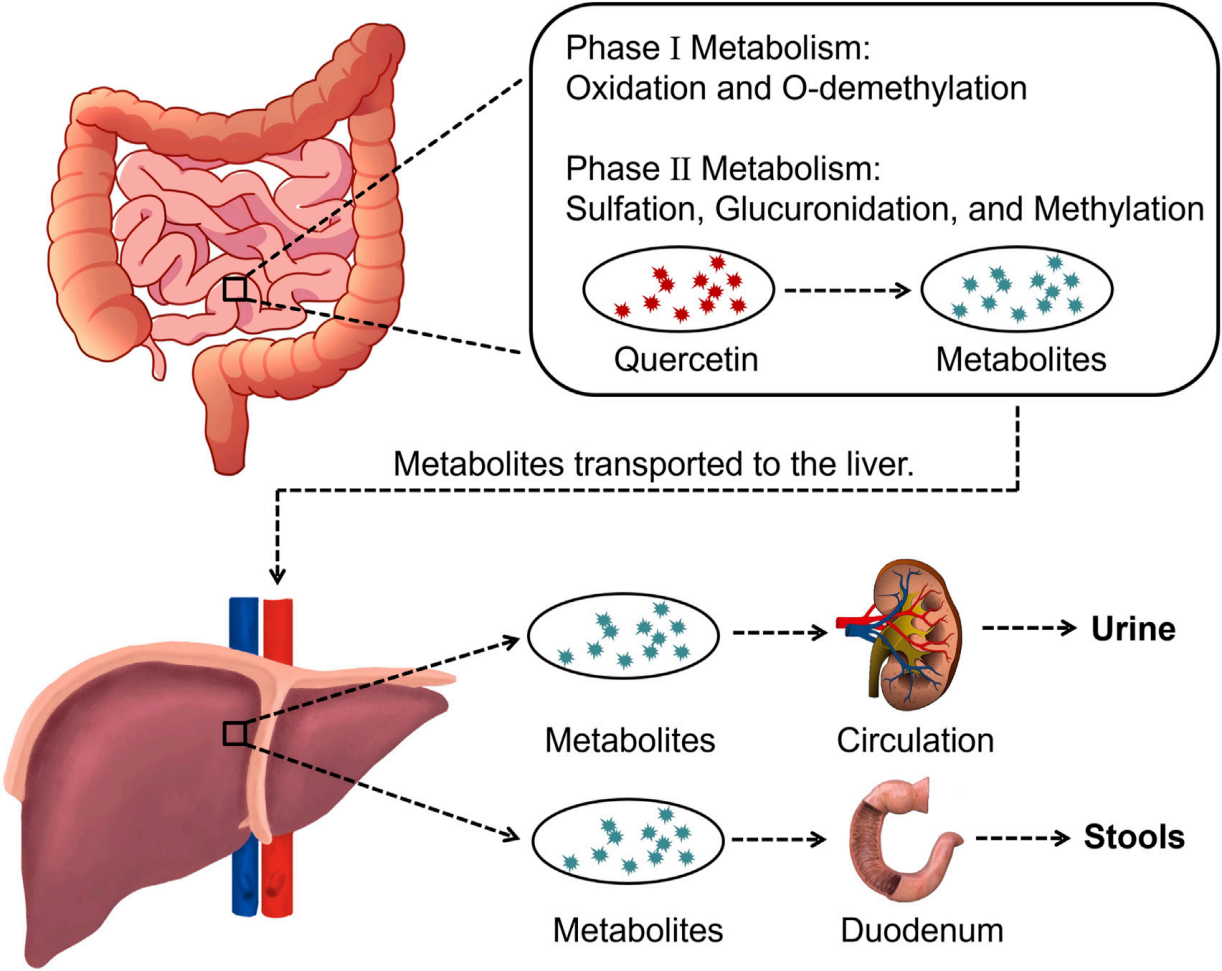
Overview of quercetin metabolization in the body.

Pomegranate peel also contains flavonoids such as quercetin, catechin, and kaempferol. Among them, quercetin is one of the most common flavonoids, though its content in pomegranate peel is relatively low (Kumar et al., 2022). After oral administration, quercetin is metabolized into derivatives such as O-glycosides and C-glycosides. These metabolites are absorbed in the small intestine, bind to serum albumin, and are transported to the liver, where they undergo sulfation, glucuronidation, and methylation to form stable metabolites, which are then distributed throughout the body (Figure 3) (Manach et al., 1995; Nijveldt et al., 2001; Zhang et al., 2023). Quercetin exerts antioxidant effects by directly scavenging reactive oxygen species (ROS) and chelating metal ions (Min et al., 2019; Tang et al., 2014). It also improves cardiovascular health by modulating the hsp70/ERK/PPAR signaling pathway (Oyagbemi et al., 2018; Pereira et al., 2018).

Catechin is one of the most studied flavonoids in pharmacology and has been reported to possess diverse pharmacological activities. Catechin exhibits anticancer properties through mechanisms such as inducing apoptosis, inhibiting cancer cell proliferation, arresting the cell cycle, and regulating tumor-related signaling pathways (e.g., inhibiting AKT phosphorylation) (Shikha et al., 2024; Shukla et al., 2018; Sun et al., 2020). Manikandan et al. utilized HCT 15, HCT 116 (human colon adenocarcinoma cells), and Hep G-2 (human laryngeal carcinoma cells) as experimental models to investigate the synergistic anticancer activity of curcumin and catechin (Manikandan et al., 2012). The results indicated that both curcumin and catechin alone, as well as their combination, effectively inhibited the proliferation of HCT 15, HCT 116, and Hep G-2 cells. However, when combined, the cytotoxicity was more pronounced, significantly disrupting the monolayer of the cells. This suggests a synergistic effect between catechin and other anticancer agents like curcumin, enhancing the suppression of cancer cells. Notably, HCT 15, HCT 116, and Hep G-2 cells exhibited significant pathological differences, making it difficult to universally interpret the applicability of the synergistic effect. Furthermore, the study did not validate the shared targets of curcumin and catechin through molecular docking or protein interaction experiments. Therefore, future research should strengthen these aspects to clarify their molecular mechanisms. Additionally, catechin is an effective molecule for treating inflammation. It exerts anti-inflammatory effects by inhibiting the gene expression of pro-inflammatory cytokines such as IL-1α, IL-1β, IL-6, and IL-12p35, downregulating the activity of inflammatory enzymes like iNOS and COX-2, and suppressing key factors in inflammation signaling pathways such as NF-κB, FOXO3a, and SIRT1(Cheng et al., 2019). Moreover, catechin has been found to exhibit antibacterial activity against various bacterial strains by disrupting bacterial membranes, inhibiting spore and vegetative cell growth, and inducing oxidative stress (Hara-Kudo et al., 2005; Parvez et al., 2019). However, despite these studies showcasing the bioactivity of catechins, their clinical efficacy may be limited due to their low bioavailability *in vivo*.

Kaempferol is another important flavonoid metabolite found in pomegranate peel, exhibiting multiple bioactivities such as antiviral, anti-inflammatory, anti-metabolic disorder, and cardiovascular protective effects (Al-Numair et al., 2015; Hussain et al., 2024; Huwait et al., 2022; Zhuang et al., 2017). In terms of antiviral effects, kaempferol effectively inhibits hepatitis B virus activity by suppressing the synthesis of hepatitis B surface antigen and hepatitis B e antigen (Parvez et al., 2022). Studies have revealed that increasing kaempferol concentration significantly reduces intracellular viral DNA levels (Li et al., 2008). Additionally, kaempferol demonstrates neuroprotective properties in neurodegenerative diseases. In Alzheimer’s disease models of *Drosophila* expressing wild-type human amyloid-beta 42, kaempferol alleviates symptoms by reducing oxidative stress in the brain (Han et al., 2019). Li et al. induced Parkinson’s disease in C57BL/6 mice by injecting MPTP (neurotoxin) and treated the mice with different doses (25, 50, and 100 mg/kg) of kaempferol for 14 consecutive days prior to MPTP injection (Li and Pu, 2011). The results showed that kaempferol could effectively protect dopaminergic neurons by increasing the activity of endogenous antioxidants (such as superoxide dismutase and glutathione peroxidase) and decreasing malondialdehyde levels. These studies confirmed the neuroprotective effects of kaempferol *in vitro* models. However, the MPTP model primarily simulates the motor symptoms of Parkinson’s disease but does not fully replicate pathological features such as α-synuclein aggregation. Additionally, current research has not compared the efficacy of kaempferol with commonly used neuroprotective drugs. Future studies could consider using α-synuclein overexpressing transgenic mice to better simulate the pathological characteristics of Parkinson’s disease, combined with comparative experiments, to further assess the relative efficacy and application potential of kaempferol in neuroprotection.

Granatin A and Granatin B are two flavonoids predominantly found in pomegranate peel, differing mainly in the type and number of glycosides they contain. Their content in pomegranate peel is relatively low, with yields of 0.003% and 0.013%, respectively, obtained *via* fractionation techniques such as column chromatography (Lee et al., 2010). Despite structural differences, Granatin A and Granatin B generally exhibit similar bioactivities, primarily in anti-inflammatory and anticancer applications. Research has shown that they selectively inhibit microsomal prostaglandin E synthase-1 expression in non-small cell lung cancer A549 cells, downregulate tumor necrosis factor-α, inducible nitric oxide synthase, and anti-apoptotic factor B-cell lymphoma 2, and induce apoptosis in A549 cells (Toda et al., 2020). Additionally, these metabolites effectively suppress NO production and nitric oxide synthase expression in RAW 264.7 macrophages, thereby exerting anti-inflammatory effects (Lee et al., 2010). These findings highlight the potential applications of Granatin A and Granatin B in anti-inflammatory and anticancer research. However, due to their low yields, this may limit their future application in the biomedical field.

### 3.3 Protocatechuic acid

Phenolic acids are an important subclass of polyphenolic metabolites, characterized by the presence of one or more hydroxyl (-OH) and carboxyl (-COOH) groups attached to a benzene ring (Sun and Shahrajabian, 2023; Wani et al., 2023). Protocatechuic acid (PCA), or 3,4-dihydroxybenzoic acid, is one of the main phenolic acids found in pomegranate peel and is also a metabolite of flavonoid metabolites such as quercetin (Azzini et al., 2010; Crozier et al., 2010; Singh et al., 2016). PCA exhibits various bioactivities, including antioxidant, anti-inflammatory, and antibacterial properties, playing an important role in the prevention and treatment of diseases such as cancer, diabetes, and Alzheimer’s disease.

**FIGURE 4:**
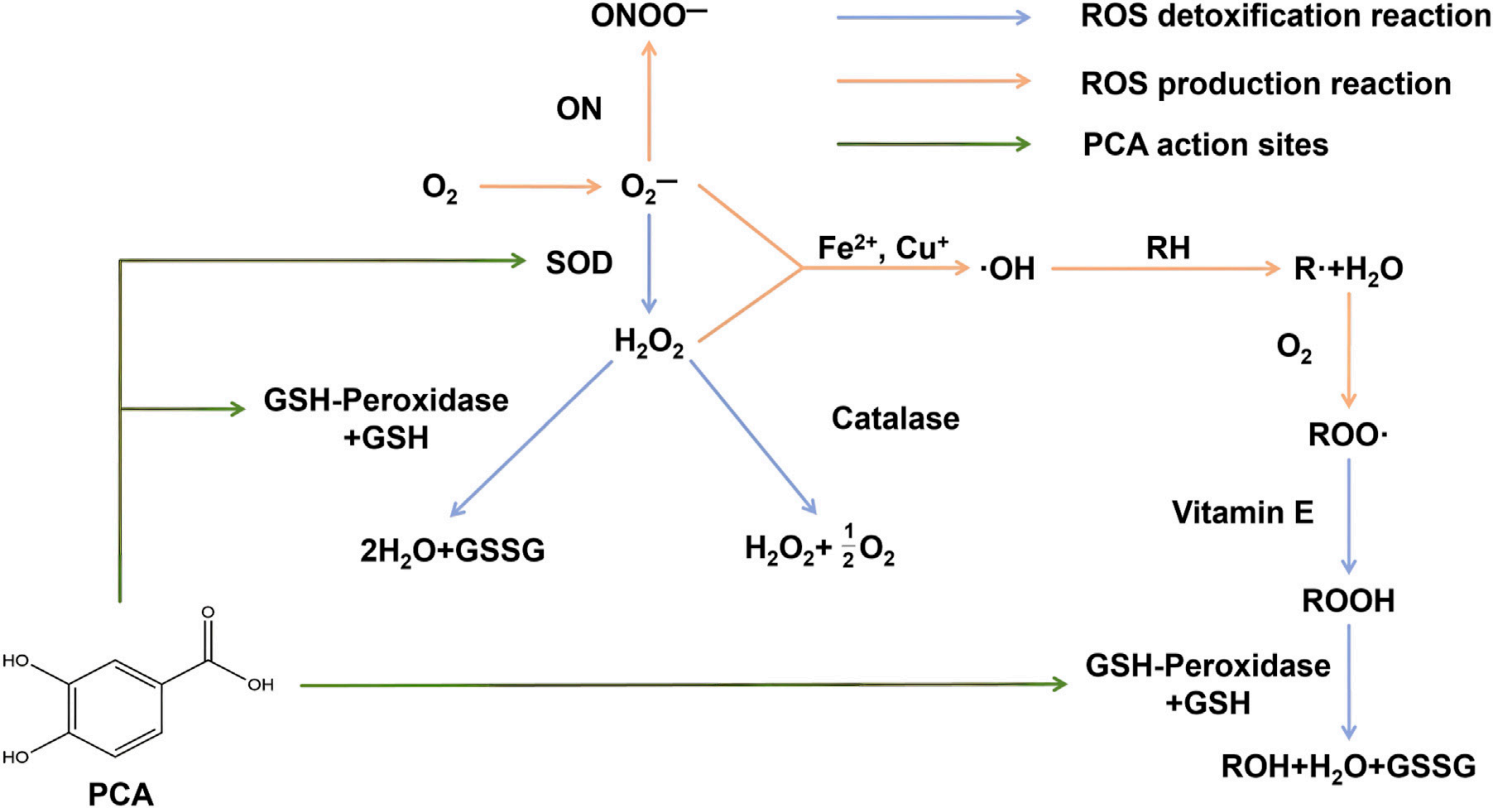
PCA can enhance the activity of SOD and GSH-PX enzymes, thereby playing a regulatory role in maintaining redox balance.

ROS are byproducts of aerobic metabolism and primarily include superoxide anion (O_2_^−^), hydrogen peroxide (H_2_O_2_), and hydroxyl radical (⋅OH). When ROS become free radicals due to unpaired electrons, if not neutralized by antioxidants in a timely manner, they can cause oxidative damage to biological macromolecules such as lipids, proteins, and DNA. This oxidative damage can lead to cellular dysfunction, aging, and the onset of various diseases (Song et al., 2020). As a natural polyphenolic metabolite, PCA exerts its antioxidant effects through multiple mechanisms. PCA enhances the activity of intracellular antioxidant enzymes, such as superoxide dismutase (SOD) and glutathione peroxidase (GSH-Px), promoting the clearance of ROS and alleviating oxidative stress in cells (Figure 4) (Cadena-Iniguez et al., 2024; Laskar and Mazumder, 2020). PCA also inhibits the Fenton reaction catalyzed by metal ions (e.g., Fe^2+^, Cu^+^), reducing the production of hydroxyl radicals and mitigating oxidative stress from the source (Shahidi and Ambigaipalan, 2015). Additionally, compared to the standard antioxidant Trolox, PCA has demonstrated stronger *in vitro* antioxidant activity in both lipid and aqueous environments (Kakkar and Bais, 2014). Therefore, with its multiple antioxidant mechanisms, PCA serves as an effective natural antioxidant, aiding in the prevention of aging and various diseases related to oxidative stress.

Inflammatory processes are regulated by various cytokines, chemokines, and inflammatory mediators. Pro-inflammatory cytokines such as TNF-α can bind to corresponding receptors and activate signaling pathways like NF-κB and MAPK, which in turn induce the expression of pro-inflammatory factors like IL-1 and IL-6, thereby exacerbating the inflammatory response (Al-Khayri et al., 2022; Hirano, 2021; Ren et al., 2020). In vitro experiments with RAW 264.7 cells, PCA was shown to inhibit the expression of TNF-α by modulating the activation of the NF-κB and MAPK pathways (Min et al., 2010). Wang et al. used AIN-93G diet containing PCA or standard AIN-93G diet to feed apoE knockout mice for 20 weeks (Wang et al., 2010). The animal experiment results showed that the PCA supplement reduced the area of aortic sinus plaques by 42%. PCA exerted anti-inflammatory effects by lowering the expression of Vascular Cell Adhesion Molecule 1 (VCAM-1) and Intercellular Adhesion Molecule 1 (ICAM-1) in the aorta, inhibiting NF-κB activity, and reducing the levels of soluble VCAM-1 and ICAM-1 in plasma, thereby inhibiting the development of atherosclerosis. These findings suggest that PCA, as a natural metabolite, can regulate inflammation-related signaling pathways, showing potential for anti-inflammatory applications and the treatment of inflammation-related diseases.

Among plant-derived active metabolites, antimicrobial potency generally decreases in the order of phenols, aldehydes, ketones, alcohols, ethers, and hydrocarbons, making polyphenolic extracts ideal candidates to replace traditional antimicrobial agents (Nguyen et al., 2018). As a polyphenolic metabolite derived from plants, PCA exhibits significant antibacterial activity, and has been shown to specifically inhibit the growth of *Pseudomonas aeruginosa*, *Listeria monocytogenes*, *Aeromonas hydrophila*, *Pasteurella multocida*, *Escherichia coli*, and *certain Salmonella strains*, while also reducing the pathogenicity of other bacteria such as *Proteus mirabilis* (Aldulaimi, 2017; Alvarado-Martinez et al., 2020; Bernal-Mercado et al., 2020; López-Romero et al., 2018; Torzewska and Rozalski, 2014). Research has indicated that the antimicrobial mechanism of PCA primarily involves disrupting bacterial membrane integrity, interfering with bacterial metabolism, and inhibiting biofilm formation (Tian L. et al., 2022; Wu et al., 2022; Yuan et al., 2024). Additionally, when used in combination with other antibiotics, PCA demonstrates synergistic effects, further enhancing its antibacterial efficacy (Fifere et al., 2022). These multiple mechanisms make PCA a promising candidate for antimicrobial applications.

Furthermore, PCA also possesses anti-apoptotic and anti-tumor activities. Abdelrahman et al. induced chronic liver injury and hepatic encephalopathy in male BALB/c mice (7–9 weeks old) through intraperitoneal injection of thioacetamide (200 mg/kg, 3 times per week) (Abdelrahman and El-Tanbouly, 2022). Starting from the fourth week, the mice were treated with oral PCA (100 mg/kg and 150 mg/kg). The results showed that PCA significantly restored the normal histological structure of the liver and brain tissues of the treated mice by downregulating the expression of Caspase-3 in the liver and the expression of tumor protein p53 in both the liver and brain, showing a significant anti-apoptotic effect. However, the PCA dosage used in the study was much higher than the normal dietary intake, which creates a gap in the clinical translational application. PCA’s anti-apoptotic mechanisms are closely related to its regulation of the abnormal expression of mitochondrial pathway-related proteins such as Bcl-2-associated X protein and cytochrome c, and its ability to modulate the Nrf2/p62 pathway, reducing oxidative stress and apoptotic signaling in cells (Zhao et al., 2021). In addition to its anti-apoptotic effects, PCA exerts anti-tumor activity through various mechanisms. PCA significantly reduces the expression of matrix metalloproteinase 2 (MMP2) by inhibiting signal transduction in the RhoB/PKCε and Ras/Akt pathways, thereby preventing tumor cell migration and invasion (Cadena-Iniguez et al., 2024; Lin et al., 2011). PCA also inhibits the Heme Oxygenase-1 (HO-1) mediated activation of p21, reducing colorectal cancer cell viability and inducing apoptosis (Acquaviva et al., 2021). Moreover, when combined with anticancer drugs such as 5-fluorouracil, PCA enhances the efficacy of chemotherapy while minimizing drug-induced side effects (Motamedi et al., 2020). Through these various mechanisms, PCA shows promising anti-tumor activity and potential for use in combination with other anticancer drugs.

Gallic Acid is one of the major phenolic acid metabolites in pomegranate, particularly abundant in the peel. The concentration of gallic acid in every 100 g of pomegranate peel is approximately 128 mg (Singh et al., 2023). Gallic acid exhibits various biological properties, with its antioxidant capacity being the most notable (Wianowska and Olszowy-Tomczyk, 2023). For instance, gallic acid is used as a standard antioxidant in the DPPH method (Pyrzynska and Pękal, 2013), and its antioxidant activity has been shown to surpass common antioxidants such as uric acid, water-soluble vitamin C, and ascorbic acid (Antolovich et al., 2002). Methyl gallate, an ester derivative of gallic acid, also exhibits significant antioxidant activity (Liang et al., 2023). Regarding anti-inflammatory activity, gallic acid can inhibit inflammation induced by lipopolysaccharides, including the release of nitric oxide, prostaglandin E2, interleukin-6, and cyclooxygenase-2 (BenSaad et al., 2017; Marinov et al., 2019). Its anti-inflammatory mechanism may involve the suppression of pro-inflammatory signaling pathways such as NF-κB, thereby alleviating inflammatory responses and exerting protective effects (Zhu et al., 2019). In terms of antitumor activity, Sun et al. investigated the effect of gallic acid on the HepG2 and SMMC-7721 human hepatocellular carcinoma (HCC) cell lines (Sun et al., 2016). The results showed that gallic acid effectively inhibited the growth of liver cancer cells, with IC50 values of 28.5 ± 1.6 μg/mL and 22.1 ± 1.4 μg/mL, respectively. Gallic acid suppressed the proliferation of both HepG2 and SMMC-7721 human liver cancer cells in a time- and dose-dependent manner by activating caspase-3 and caspase-9 and promoting the generation of reactive oxygen species. For antibacterial activity, gallic acid can inhibit the growth of pathogenic bacteria such as *Escherichia coli*, *S. aureus*, and *Campylobacter jejuni* by reducing membrane permeability and regulating pH levels (Kang et al., 2008; Sarjit et al., 2015). Moreover, gallic acid demonstrates anti-apoptotic activity by counteracting apoptosis mediators such as 6-hydroxydopamine (Erol-Dayi et al., 2012). Similarly, vanillic acid, which contains a phenolic hydroxyl group, can scavenge ROS and exhibits antioxidant properties (Parvin et al., 2020). Additionally, numerous studies have highlighted the pharmacological potential of vanillic acid in cardiovascular diseases (Khoshnam et al., 2018), inflammatory pain (Calixto-Campos et al., 2015), and asthma (Bai et al., 2019).

## 4 Biomedical applications of PPE

Pomegranate peel is rich in bioactive metabolites, exhibiting multiple health benefits, including antioxidant, anti-inflammatory, antibacterial, anti-apoptotic, and anticancer effects. The bioactive substances and biocompatibility of pomegranate peel make it an ideal candidate for the preparation of biomedical materials such as nanodrug carriers and hydrogels, offering promising applications for PPE in drug delivery, tissue repair, and regenerative medicine. Specifically, the antioxidant and anti-inflammatory properties of PPE help mitigate oxidative stress and inflammation caused by biomedical materials during their application, thereby enhancing the stability and therapeutic efficacy of these materials. Furthermore, PPE’s inherent antibacterial activity provides infection resistance to biomedical materials, significantly improving their potential applications in wound healing and infection control. For instance, in the context of tissue engineering scaffolds, PPE can significantly enhance the bioactivity and antioxidant properties of scaffolds, thereby promoting cell adhesion, proliferation, and tissue repair. The bioactive molecules in PPE regulate cellular signaling pathways, effectively improving the inflammatory microenvironment and further accelerating tissue regeneration. Consequently, PPE demonstrates significant advantages in drug delivery, tissue repair, and regenerative medicine, paving the way for the development of novel biomedical materials with multifunctional properties.

### 4.1 Nanodrug carriers

Drug carriers can improve therapeutic outcomes by altering the pharmacokinetics and biodistribution of conventional drugs (bare drugs) or by acting as reservoirs and slow-release systems for conventional drugs (Cheng et al., 2021; Guo et al., 2021; Tenchov et al., 2021). To summarize, drug carriers must exhibit good biocompatibility and efficiently and safely deliver drugs to target sites. By providing sustained-release characteristics, these carriers can prolong drug action, reduce dosing frequency, and improve therapeutic outcomes. Nanodrug carriers can be broadly categorized into three types based on the materials used for their preparation: inorganic nanodrug carriers, lipid-based nanodrug carriers, and polymer-based nanodrug carriers.

Inorganic nanodrug carriers are typically made from metals, metal oxides, or carbon-based materials (such as gold nanoparticles, silver nanoparticles, silica nanoparticles, carbon nanotubes, *etc.*), which often possess unique optical, electrical, and magnetic properties, making them highly advantageous in cancer treatment, imaging, diagnostics, and other fields (Du et al., 2023; Khan et al., 2022; Khizar et al., 2023; Raza et al., 2021; Shang et al., 2022; Song et al., 2021). Taherian et al. prepared nanoparticles BPPE-CCMNPs containing PPE through a two-step precipitation method and evaluated their anticancer capabilities (Taherian et al., 2021). These nanoparticles, with a size of 37 nm, demonstrated stability in biological environments and showed a favorable release profile in tumor-simulating conditions. Cytotoxicity results indicated that these nanoparticles were toxic to cancer cells but non-toxic to normal cells, suggesting better prospects compared to chemotherapy drugs. Additionally, PPE can serve as a reducing agent and stabilizer for the green synthesis of gold and silver nanoparticles, which are used in antibacterial and cancer treatments (Fu et al., 2023; Ghobadi et al., 2024; Khan et al., 2021). Although researchers have developed various PPE-based inorganic nanodrug carriers using different methods, the potential of inorganic materials, particularly in exploring their optical, electrical, and magnetic properties, has not been fully harnessed. For instance, gold and silver nanoparticles hold significant promise in photothermal therapy and imaging. Further research is needed to combine the physical properties of these nanomaterials with the bioactivity of PPE to achieve dual or multiple therapeutic effects.

Lipid-based nanodrug carriers, composed of natural or synthetic lipid molecules (such as phospholipids, cholesterol, *etc.*), can spontaneously form bilayer structures called liposomes (Liu P. et al., 2022; Nsairat et al., 2022). Lipid-based nanodrug carriers can efficiently encapsulate both hydrophilic and hydrophobic drugs, and they offer high drug-loading capacity and good stability (Albertsen et al., 2022). Okumus et al. used maltodextrin and soybean lecithin to encapsulate PPE, with Zeta potentials of −18.05, −15.51, and −57.6 for PPE and the encapsulated nanoparticles, respectively, indicating that liposomal encapsulation can enhance PPE’s stability (Okumus et al., 2021). Wang et al. prepared lipid carriers containing PPE using a warm microemulsion technique, and the nano-liposomes demonstrated sustained release for 12 h, with strong anti-tyrosinase activity (Tokton et al., 2014). However, despite the advantages of lipid-based nanodrug carriers, they still face challenges related to solubility, stability, and aggregation (Andishmand et al., 2023). Future efforts could focus on optimizing formulations or modifying the surface of nanoparticles to enhance the application potential of PPE in cancer therapy and other fields.

Polymer-based nanodrug carriers are another widely used carrier system, typically made from synthetic or natural polymer materials such as polylactic acid (PLA), polyethylene glycol (PEG), and poly (D,L-lactic-co-glycolic acid) (PLGA) (De et al., 2022; Dou et al., 2020; Du et al., 2020; Ibrahim et al., 2022; Shi et al., 2022). These carriers possess tunable physicochemical properties and excellent biocompatibility, allowing for sustained drug release (Arkaban et al., 2022). Shirode et al. developed PLGA-PEG nanoparticles loaded with PPE (Shirode et al., 2015), while Soltanzadeh et al. employed chitosan nanoparticles to encapsulate PPE (Soltanzadeh et al., 2021), both nanoparticles exhibited good anticancer and antibacterial activity, along with sustained drug release. However, one major advantage of polymer-based nanocarriers is their ability to achieve targeted delivery to specific cells or tissues through surface functionalization (Ghosh and Biswas, 2021; Shi et al., 2021), research on PPE-based polymer nanocarriers remains largely unexplored.

In recent studies, many nano-drug carriers have been prepared using PPE. These studies highlight that PPE’s biocompatibility and bioactivities—such as antibacterial and anticancer properties—endow nano-drug carriers with excellent compatibility and the ability to inhibit bacterial and cancer cell growth. However, these studies still face numerous challenges. Inorganic nano-drug carriers exhibit various properties depending on the materials used. For instance, metallic nanoparticles can be utilized to prepare photothermal and imaging systems for photodynamic therapy (Ouyang et al., 2021; Xing et al., 2018; Zhan et al., 2024), offering unique advantages in cancer treatment. Current research has yet to fully explore and leverage these properties. Combining these physical properties with the bioactivities of PPE could lead to more effective therapies. Additionally, targeting is a critical factor in evaluating the performance of drug carrier systems. Most anticancer nano-drug carriers prepared with PPE rely on the enhanced permeability and retention (EPR) effect for passive targeting, which has limitations, such as uneven distribution within tumor tissues or incomplete drug release (Gawali et al., 2023; Takakura and Takahashi, 2022). Future studies could explore strategies such as ligand or antibody modifications to enhance active targeting, ensuring precise delivery of carriers to target regions and effective release of drugs to maximize PPE’s bioactivities.

### 4.2 Hydrogels

Hydrogels are a class of soft polymer materials with a three-dimensional network structure that can absorb large amounts of water or biological fluids while maintaining their shape and structure (Norahan et al., 2023; Qiao et al., 2023; Shao et al., 2023). Due to their good biocompatibility, tunable mechanical properties, and ability to mimic natural tissues, hydrogels are widely used in wound dressings and tissue engineering. The polyphenolic metabolites in PPE have antibacterial and antioxidant properties, making it an ideal additive for the preparation of functional hydrogels.

**FIGURE 5:**
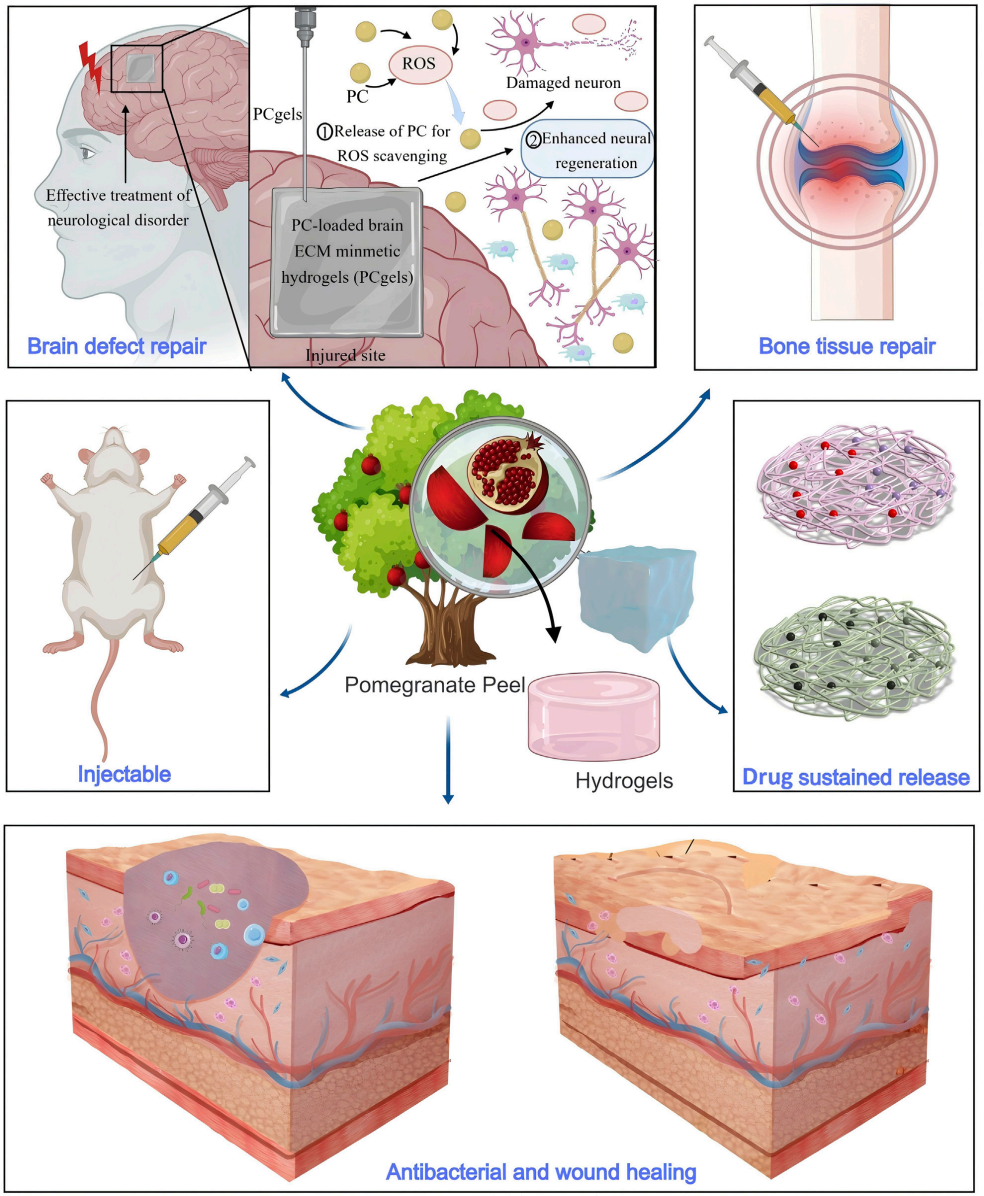
Hydrogels containing PPE exhibit properties such as injectable, antibacterial activity, and drug sustained release, making them suitable for applications in wound healing, brain defect repair, and bone tissue repair.

In terms of wound dressings, hydrogels containing PPE offer significant advantages (Figure 5). First, the polyphenolic metabolites in PPE effectively inhibit bacterial growth, reducing the risk of infection. Ul-Islam et al. developed an antibacterial wound dressing based on bacterial cellulose loaded with PPE (Ul-Islam et al., 2023). This dressing demonstrated good water absorption and retention capabilities, with excellent antibacterial performance, achieving 100% inhibition of *S. aureus* growth and 50% inhibition of *Escherichia coli*. Secondly, PPE can accelerate wound healing by regulating signaling pathways. Savekar et al. prepared a hydrogel film from PPE, citric acid, and β-cyclodextrin-carboxymethyl cassava starch, which could prevent wound infection and accelerate the healing process (Savekar et al., 2024). This film is biodegradable and exhibits pH-dependent ellagic acid release within 24 h. The released ellagic acid promotes cell division, proliferation, and migration to the wound site by inhibiting Glycogen Synthase Kinase-3 Beta and Casein Kinase 1, thereby promoting wound healing.

In the field of regenerative medicine, PPE-based hydrogels also show broad application potential. Hosseini et al. developed an injectable nanocomposite hydrogel based on polyethylene glycol and chitosan for sustained PPE release (Hosseini et al., 2023). This hydrogel can steadily release PPE for up to 360 h and effectively repair bone injuries by promoting osteoblast differentiation. Similarly, Garcia et al. developed a hydrogel for bone tissue repair, loaded with extracts from pomegranate peel, grape seed, and jabuticaba peel. The study showed that only the hydrogel containing PPE could form an inhibition zone around *S. aureus*, demonstrating significant antibacterial activity (Garcia et al., 2021). In addition to bone tissue repair, Ju et al. prepared an injectable hydrogel using PPE, hyaluronic acid, and collagen for brain defect repair (Ju et al., 2023). This hydrogel could be precisely injected through a 23-gauge needle (inner diameter 337 μm) to adapt to the deep folds of brain tissue and specific defect areas. The loaded PPE not only exhibited ROS scavenging properties but also promoted neuronal regeneration and differentiation, effectively repairing brain tissue defects.

Hydrogels, with their three-dimensional network structure, provide high hydration capacity, effectively maintaining a moist wound environment and promoting healing, making them ideal wound dressings. Hydrogels prepared with PPE can exhibit antibacterial and antioxidant properties, making them highly effective in preventing infections and accelerating wound healing. The polyphenolic metabolites in PPE effectively inhibit bacterial growth, reducing the risk of wound infection, while its antioxidant properties alleviate oxidative stress and promote cell repair. Additionally, PPE can regulate cellular signaling pathways, facilitating cell proliferation and migration to accelerate the wound healing process. However, despite the significant antibacterial and wound-healing effects of PPE-based hydrogels, challenges remain. For example, the mechanical properties and durability of hydrogels can sometimes be weak, which may affect their performance in long-term applications. Future research could explore combining PPE with cross-linking agents such as polyvinyl alcohol or extracellular matrix metabolites to enhance the mechanical properties and stability of hydrogels in complex wound environments (Chen et al., 2024; Morton et al., 2023). Furthermore, improving the controlled release mechanisms and structural design of hydrogels to introduce responsiveness to stimuli such as temperature and pH (Haidari et al., 2021; Yu et al., 2024) could enhance their effectiveness and reliability in long-term treatments.

### 4.3 Tissue engineering scaffolds

Tissue engineering scaffolds are three-dimensional porous solid materials that typically possess high mechanical strength. Their main purpose is to provide a temporary physical framework for damaged tissues, facilitating cell attachment, migration, and proliferation through their porous structure, thereby offering the necessary physical support for the growth of new tissue (Arif et al., 2022; Janmohammadi et al., 2023; Zhang et al., 2022). PPE promotes osteoblast differentiation and proliferation, inhibits osteoclast activity, and exhibits significant anti-inflammatory and antibacterial properties, enabling it to accelerate tissue repair and reduce inflammatory responses. With these advantages, PPE has been utilized in the design of tissue engineering scaffolds.

**FIGURE 6:**
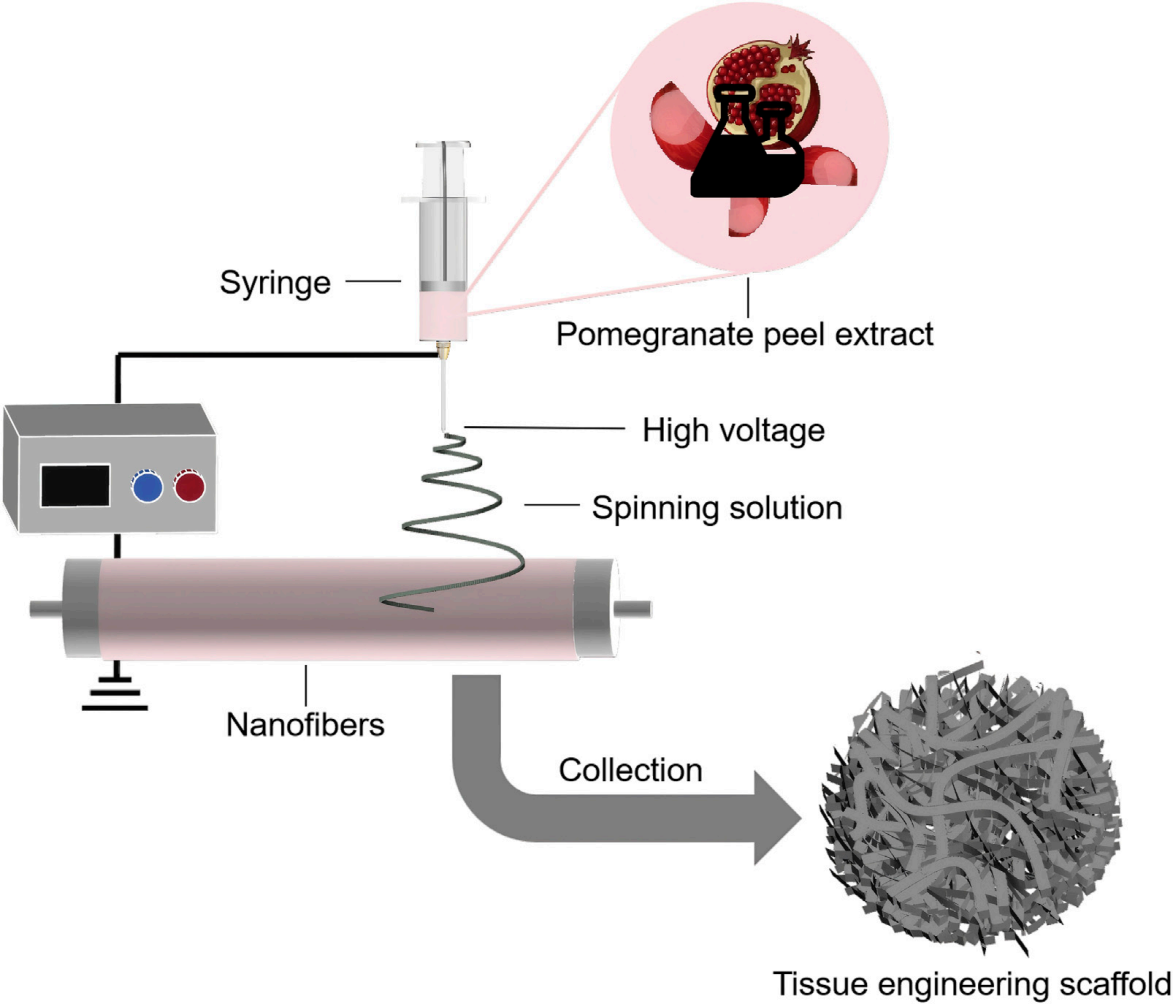
Schematic illustration of the preparation of PPE-containing tissue engineering scaffolds using electrospinning technology.

PPE has been shown to promote osteoblast differentiation and proliferation, inhibit osteoclast activity, and exhibit significant anti-inflammatory and antibacterial properties, accelerating bone tissue repair and reducing inflammatory responses (Bahtiar et al., 2014; Kim et al., 2009; Siddiqui and Arshad, 2014; Spilmont et al., 2015). Owing to these advantages, PPE has been widely applied in the design of scaffolds for bone tissue repair (Figure 6). Sadek et al. used electrospinning technology to prepare a tissue engineering scaffold containing PPE, which demonstrated a nearly 96% improvement in antioxidant activity compared to scaffolds without PPE, along with significant enhancements in osteoblast attachment and proliferation (Sadek et al., 2021). Oliveira et al. further evaluated the effect of polymer scaffolds with PPE on repairing mandibular defects in rats (de Oliveira et al., 2024). The experiment involved three groups: no graft, polymer scaffolds with Brazilian palm resin, and polymer scaffolds with PPE. The average percentages of bone volume formed in these groups were 17.17% ± 2.68%, 27.45% ± 1.65%, and 34.07% ± 0.64% (mean ± standard deviation), with the PPE-containing polymer scaffolds showing the best bone repair ability. Additionally, Karabulut et al. used 3D printing technology to create dental membrane scaffolds containing PPE, and their study showed that the addition of PPE significantly enhanced the scaffold’s biocompatibility and antibacterial activity (Karabulut et al., 2023).

Tissue engineering scaffolds based on PPE have achieved notable results in the field of bone tissue repair, owing to their exceptional bioactivity. Currently, tissue engineering scaffolds have been widely applied in the repair of soft tissue, cardiovascular systems, and skin (Chen et al., 2023; Roacho-Perez et al., 2022; Weems et al., 2021). By incorporating drugs or enabling controlled release of gases (e.g., oxygen, nitrogen, *etc.*) (Augustine et al., 2023; Li et al., 2020; Mo et al., 2023), these scaffolds can provide more personalized therapeutic effects. Future studies could explore the potential of PPE in repairing other types of tissues and facilitating combination therapies.

### 4.4 Other biomedical applications

In addition to being an important component of nanodrug carriers, hydrogels, and tissue engineering scaffolds, PPE is also widely applied in fields such as skin repair in the form of ointments and films. In skin repair studies, Luo et al. successfully developed a pomegranate polyphenol ointment and applied it to a scarred rat model (Luo et al., 2022). The results showed that the ointment significantly reduced the damaged area on the rats’ skin and decreased the expression levels of proteins such as Alpha-Smooth Muscle Actin, Phosphorylated Extracellular Signal-Regulated Kinase 1/2, Phosphorylated Janus Kinase 2, Phosphorylated Signal Transducer and Activator of Transcription 3, and Phosphorylated Protein Kinase B. These effects effectively alleviated inflammatory responses, inhibited scar formation, and promoted the skin repair process. Similarly, Hayouni et al. prepared a skin repair ointment using PPE, which demonstrated outstanding antioxidant and antibacterial activities. The ointment significantly accelerated wound contraction and epithelialization in guinea pigs and exhibited potent antibacterial effects against *P. aeruginosa*, *S. aureus*, and *Escherichia coli* (Hayouni et al., 2011). Additionally, Barbalinardo et al. combined PPE with natural silk fibroin to create a transparent, flexible, and bioactive film (Barbalinardo et al., 2022). This film, when applied to skin repair, effectively reduced oxidative stress-induced cell damage and promoted skin regeneration and repair. Beyond skin repair, Jebahi et al. developed a medical dressing using PPE for the treatment of gastric ulcers (Jebahi et al., 2022). In a rabbit gastric ulcer model, the dressing exhibited high blood compatibility (with a hemolysis rate of only 0.42%) and significant antibacterial activity against *S. aureus* and *Helicobacter pylori*, with inhibition rates of 84.62% and 88.41%, respectively, after 18 h of contact. The dressing effectively reduced the incidence and severity of gastric tissue lesions in rabbits, demonstrating excellent anti-ulcer effects. Furthermore, Silva developed an oral adhesive ointment using PPE to improve canine oral hygiene. This ointment showed significant antibacterial activity against oral microbiota in Labrador dogs (Silva et al., 2020). In summary, PPE, with its excellent antioxidant, antibacterial, and anti-inflammatory properties, provides innovative solutions for skin repair, gastric ulcer treatment, and oral care.

With the continued advancement of biomedical materials research, more natural biomaterials with favorable bioactivity are being developed and applied in emerging fields such as microneedle patches and medical adhesives (Ding et al., 2024; George and Suchithra, 2019; Medlej et al., 2022; Ryall et al., 2022). Although there are currently no studies on the application of PPE in these areas, its natural bioactivity and good biocompatibility suggest tremendous application potential. Future research could focus on further developing PPE for use in microneedle patches, tissue adhesives, and other biomedical materials, opening up new directions for research and application.

## 5 Conclusion

PPE is rich in bioactive metabolites such as ellagic acid, phenolic acids, and flavonoids, which possess multiple functions including antioxidant, anti-inflammatory, antimicrobial, anti-apoptotic, and cell regeneration-promoting properties. These qualities present a wide range of potential applications in biomedical fields such as nanoparticle drug delivery systems, hydrogels, and tissue engineering scaffolds. Through various preparation techniques, PPE can be effectively integrated into medical materials, significantly enhancing their biocompatibility and functionality, promoting wound healing and tissue regeneration, and showing potential in anticancer therapy. The combination of natural extracts with modern technologies not only promotes the development of green biomaterials but also provides new avenues for efficient and safe treatments. However, further research is needed to address issues such as long-term stability, precise delivery, and the mechanisms of action of PPE. In the future, its application techniques should be optimized, and its clinical therapeutic potential should be further explored to achieve broader medical applications.
